# Human adenovirus serotype 5 infection dysregulates cysteine, purine, and unsaturated fatty acid metabolism in fibroblasts

**DOI:** 10.1096/fj.202402726R

**Published:** 2025-03-07

**Authors:** Bailey‐J C. Sanchez, Rudy M. Ortiz, Juris A. Grasis

**Affiliations:** ^1^ Quantitative and Systems Biology, School of Natural Sciences University of California Merced California USA

**Keywords:** adenovirus, fibroblasts, mass spectrometry, metabolomics

## Abstract

Viral infections can cause cellular dysregulation of metabolic reactions. Viruses alter host metabolism to meet their replication needs. The impact of viruses on specific metabolic pathways is not well understood, even in well‐studied viruses, such as human adenovirus. Adenoviral infection is known to influence cellular glycolysis and respiration; however, global effects on overall cellular metabolism in response to infection are unclear. Furthermore, few studies have employed an untargeted approach, combining emphasis on viral dosage and infection. To address this, we employed untargeted metabolomics to quantify the dynamic metabolic shifts in fibroblasts infected with human adenovirus serotype 5 (HAdV‐5) at three dosages (0.5, 1.0, and 2.0 multiplicity of infection [MOI]) and across 4 time points (6‐, 12‐, 24‐, and 36‐h post‐infection [HPI]). The greatest differences in individual metabolites were observed at 6‐ and 12‐h post‐infection, correlating with the early phase of the HAdV‐5 infection cycle. In addition to its effects on glycolysis and respiration, adenoviral infection downregulates cysteine and unsaturated fatty acid metabolism while upregulating aspects of purine metabolism. These results reveal specific metabolic pathways dysregulated by adenoviral infection and the associated dynamic shifts in metabolism, suggesting that viral infections alter energetics via profound changes in lipid, nucleic acid, and protein metabolism. The results revealed previously unconsidered metabolic pathways disrupted by HAdV‐5 that can alter cellular metabolism, thereby prompting further investigation into HAdV mechanisms and antiviral targeting.

## Graphical abstract

Human adenovirus serotype 5 was used to infect HEK293 fibroblasts at low MOI (0.5, 1.0, and 2.0). Infected cells were interrogated by gas chromatography time‐of‐flight mass spectrometry (GC‐TOF‐MS) at 6, 12, 24, and 36 h post‐infection. The total metabolites were evaluated using an untargeted analysis of primary carbons. The results indicate a decrease in cysteine, guanine, and unsaturated fatty acid metabolism, while there was an increase in adenosine metabolism. These results reveal the underappreciated metabolic pathways affected by HAdV‐5 infection in fibroblasts and confirm the usefulness of untargeted metabolomics approaches to determine global metabolism in infected cells.

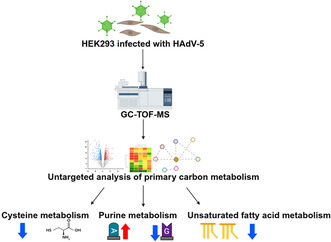

## 1 INTRODUCTION

Cellular metabolism encompasses the chemical reactions necessary for cellular livelihood. These metabolic reactions, often presumed to be successive sequences, are multidirectional, with alterations in one pathway potentially impacting multiple pathways.^1^ Different cell types in the body yield a diverse array of functions, and one cell type may possess a different metabolic landscape compared to another. Fibroblasts, which are normally quiescent cells derived from the mesoderm, are versatile in their functionality and adaptability. Metabolically, fibroblast activation is coupled with reprogramming, including upregulation of aerobic glycolysis and glutaminolysis.^2^, ^3^ Fibroblast metabolism has also been targeted for therapeutic purposes. For instance, a recent study showed that inhibition of the glycolytic enzyme 6‐phosphofructo‐2‐kinase/fructose‐2,6‐bisphosphatase 3 (PFKFB3) ameliorated pulmonary fibrosis in mice.^3^ When exposed to agonists, including viruses, fibroblasts respond with massive growth and proliferation, leading to the production of extracellular matrix material.^4^, ^5^ Similarly, fibroblast metabolism is likely to be altered by viral infections.

Given that viruses are obligate intracellular parasites that are only capable of replicating within an infected host cell, they must co‐opt multiple host pathways to enhance replication, including cellular metabolism. This cellular takeover provides the virus with the substrates and energy required for successful replication.^6^, ^7^, ^8^ Studies have attempted to examine these interactions, with early evidence tracing back to the early 1970s, when investigators became interested in the shift of cellular priority toward glycolysis and glutaminolysis, as opposed to the energetically favorable oxidative phosphorylation in the mitochondria.^9^ The prominent aspect of this change in metabolism is that as the uptake of cellular glucose increases, the output of lactate increases. It is important to note that although not nearly as much ATP is generated compared to normal oxidative phosphorylation, the ATP that is rapidly made can still fuel important cellular processes, including corresponding metabolic pathways.^10^ In the last decade, with the development of metabolomic platforms, metabolite libraries, and analytical tools, investigations into multiple viral species and global cellular metabolism have increased.^11^, ^12^, ^13^ Despite these advancements, targeted approaches are often used, including a focus on alterations to glycolysis and respiration, disregarding additional components of overall cellular metabolism.

Human adenoviruses (HAdVs) are double‐stranded DNA viruses capable of infecting the GI tract, kidneys, ocular regions, and respiratory tract.^14^ Currently, there are approximately 50 specific types of HAdVs across seven species termed A‐G, each possessing extensive tissue tropism, which can be attributed to the presence of specific protein targets, primarily coxsackie and adenovirus receptor (CAR), and membrane cofactor proteins, such as CD46.^15^, ^16^, ^17^ HAdVs possess a capsid, comprised of three major proteins: hexon, penton base, and fiber. During binding with the target cell, the penton base, with the presence of its Arg‐Gly‐Asp (RGD) motif, facilitates interaction with the αvβ integrins present in the cell surface.^18^ This is then coupled with the fiber protein binding to the target receptor, facilitating internalization into the cell. After escaping from their endosomal compartment, HAdVs are trafficked to the nucleus, mediated by microtubules and motor proteins, of which dynein and kinesin are principally involved. Fluorescence microscopy (FM) studies examining species C HAdV infection of epithelial cells have shown attachment and cellular entry within 5 min, reaching the cytosol within 15 min.^19^, ^20^ Once their DNA is deposited in the targeted nucleus, early gene expression begins 1–2 h post‐infection with the expression of the E1A gene and maximum early gene expression occurring at about 6 h post‐infection.^21^, ^22^, ^23^, ^24^, ^25^ E1A only requires transcription factors to be expressed, but this expression results in a cascade of additional early genes, notably E1B, E2A, E3, and E4 regional activation, which jointly assist in immune evasion and synthesis of new viral DNA. In contrast, late‐phase gene activation is characterized by major late promoter (MLP)‐induced transcription of the major late transcription unit (MLTU), increasing L1 to L5 gene expression.^24^, ^25^, ^26^, ^27^ These late genes will direct genome packaging, promote assembly, and regulate the formation of new viral particles. Maximal gene expression of these late genes occurs at about 12 h post‐infection. Maximal protein production of HAdV early genes occurs at about 12 h post‐infection, while maximal late gene protein production occurs at about 24 h post‐infection.^28^ Virion release from infected cells begins around 24 h post‐infection and maximally produced at about 36 h, depending on the cell type.^29^

Historically, adenovirus was one of the first viral species discovered to affect cellular metabolism. Experiments conducted by Fisher and Ginsburg revealed that serotype 4 adenovirus (HAdV‐4) infection of HeLa cells increased glycolytic elements during infection.^30^ In the 1970s, serotype 5 adenovirus (HAdV‐5) was shown to increase glucose uptake and lactate production in HEp‐2 cells.^31^ HAdV pathogenicity may affect glycolytic and respiratory metabolism, even at the level of a single protein expressed by the virus. The E4ORF1 isoform has been shown to disrupt glycolytic and glutamine metabolism in vitro.^8^, ^32^ This was observed in a breast epithelial cell line, MCF10A, infected with wild‐type HAdV‐5. Recovery of glucose and oxygen consumption, along with recovery of lactate production to baseline levels, was observed in a replication‐deficient adenovirus deletion mutant lacking the entire E4 early transcription unit region. Of the five E4 ORFs individually expressed in MCF10A cells, E4ORF1 increased glucose consumption and lactate production rates, suggesting increased glycolytic metabolism. Another study reported that A549 lung epithelial cells encoding the 13S E1A isoform from HAdV displayed upregulated glycolytic genes and downregulated genes involved in cellular respiration.^33^ While these studies provide insights into the mechanisms by which HAdV manipulates host metabolism, their focus is on the manipulation of glycolytic metabolism using targeted approaches. Furthermore, in vitro studies employing metabolomic techniques to capture the global metabolic profile of HAdV infection are lacking. Methods including ^1^H‐NMR spectroscopy have been used to understand the subversion of cell metabolism from adenoviral infection.^34^, ^35^ Specifically, HEK293 and an amniocyte‐derived cell line 1G3 were infected with recombinant E1‐deleted HAdV‐5 expressing Green Fluorescence Protein (GFP) at a concentration of 5 infectious particles/mL. Exometabolome findings included glutamine as the most consumed amino acid by both cell types, acetate was consumed only by HEK293, and formate was produced more by HEK293 than by 1G3 post‐infection. One limitation of ^1^H‐NMR spectroscopy is its reduced specificity and resolution for detecting metabolites, both known and unknown, compared to mass spectrometry techniques. Furthermore, studies examining the relationship between HAdV and host metabolism, with an emphasis on both the dose and duration of infection, are also lacking.

Studies conducted on adenoviral usurping of metabolism infect at an increased viral titer, upwards of 100 viral particles per cell. However, it is important to assess the virulence of adenoviruses at lower titers, which may better reflect physiologic conditions of infection. Furthermore, knowledge of global disruption to the host metabolome by HAdV infection, on a timescale relative to the infectious cycle, is lacking. Therefore, we assessed the effects of HAdV‐5 infection on cellular metabolism by employing gas chromatography (GC) coupled with time‐of‐flight mass spectrometry (TOF‐MS), combining the infection period with multiple dosages. We hypothesized that the disruption of cellular metabolites would be proportional to an increase in viral dosage, with the greatest shift in metabolites occurring during the 6–12 h period. Our study design allowed us to characterize the dynamic metabolic responses of human fibroblasts to adenoviral infection, yielding insights into the viral‐induced shifts in metabolism from early‐onset infection to late‐phase cell death.

## 2 MATERIALS AND METHODS

### 2.1 Cell culture

Human embryonic kidney (HEK293) cells (CRL‐1573) were obtained from the American Type Culture Collection (ATCC, Manassas, VA). Only cells that tested negative for mycoplasma contamination were used for experiments. Cells were incubated in Dulbecco's Modified Eagle's Medium (DMEM) containing low glucose, phenol red, and pyruvate (Fisher Scientific), mixed with 10% fetal bovine serum (Fisher Scientific) and 1% penicillin–streptomycin (Fisher Scientific). Incubator settings were maintained at 37.0°C and 5.0%CO_2_. Cells were passaged every 2–3 days and split at 80%–90% confluency. The medium was discarded, and the wells were washed with serum‐free DMEM and treated with 0.25% trypsin–EDTA (Fisher Scientific). Once cells had visually lifted from the flask, they were transferred to a new T‐25 flask in a 1:3 ratio, with fresh, complete growth media added to adequately cover the cells. Cell counts and viability were determined using trypan blue staining and a hemocytometer. Cells used in the experiments were not used after passage 20.

### 2.2 HAdV‐5 propagation

HAdV‐5 was obtained from the American Type Culture Collection (VR‐1516). HEK293 cells were used for viral propagation to obtain a highly concentrated viral stock. For viral stock propagation, after 3 days of HAdV‐5 infection in a T‐75 flask, spent media was removed, cells were manually scraped, base media was added, and the cells were transferred to a centrifuge tube. The cells were then pelleted, and three freeze/thaw cycles were performed, in which the samples were placed in liquid nitrogen for 30 s for each cycle. The cells were then transferred to a 15 mL, 50 kDa ultracentrifuge filter unit (Fisher Scientific). The filter units were centrifuged at 2000 rpm for 20 min in a swinging‐rotor centrifuge unit (Beckman Coulter). The retained product containing the virus was then used to infect new host cells. This was repeated three more times, and the stocks were stored at −80.0°C.

### 2.3 Reverse transcription quantitative polymerase chain reaction (RT‐qPCR)

To confirm viral infection and quantify viral titers, the infected cells were subjected to RT‐qPCR. Media harboring infected cells were removed, cells were washed with serum‐free media, and cells were treated with guanidine isothiocyanate lysis buffer (TRIzol, ThermoFisher).^36^ cDNA synthesis was performed following the manufacturer's guidelines in the SuperScript III Reverse Transcriptase kit (ThermoFisher).^37^ The adenoviral genes of interest used for this analysis were E2A and E4. Primers were designed de novo using Primer‐BLAST (Primer 3 version 2.5.0) (Supporting Information Table I, ^38^). Primers were designed to span all regions of the genes and are indicated by the identifiers HuAdV5‐PanE2A and HuAdV5‐PanE4, which have been previously validated in SYBR‐based dye qPCR assays.^39^ GAPDH was used as a control gene. QPCR reactions to confirm viral infection were performed using an Eco Real‐Time PCR System (Illumina). Viral titer was achieved by using a modified SYBR‐based dye assay protocol featuring the HuAdV5‐PanE1A primer set.^40^

### 2.4 Intracellular metabolite profiling

To perform viral infections for metabolomic analyses, the cells were passaged into 6‐well plates. The cell density at the time of infection was 1.0 × 10^6^ cells/mL. Cells were infected for the following periods: 6, 12, 24, and 36 h. We chose to evaluate metabolites at these time points due to early gene expression beginning 1–2 h post‐infection^21^, ^22^, ^23^ and viral transcripts continuing to be expressed up to 36 h.^36^ Additionally, we observed cell detachment at 48 h after infection, an indicator of cell death, which we confirmed with Propidium Iodide staining. Therefore, we decided to limit our metabolism observations to 36 h, before cell death and while the cells were still metabolically active. Noninfected cells cultured for 24 h were used as a negative control. All treatment cells were serum‐starved for 24 h prior to viral infection. Cells were subjected to the following viral dosages: 0.5, 1.0, and 2.0 multiplicity of infection (MOI), and six replicates were used for each experimental condition. Cellular pellets were collected at each time point following the removal of media, washing with 2 mL of phosphate buffered saline (PBS), and subsequent cell scraping. Once collected into pre‐chilled 2 mL microcentrifuge tubes, cells were centrifuged at 1000× *g* for 5 min, excess PBS was removed, and the pellets were stored at −80.0°C. Pooled QC samples were generated by combining 10 μL of each sample. Metabolite extraction was performed using 1 mL of a one‐phase mixture of degassed isopropanol/acetonitrile/water (3:3:2) at −20°C for 5 min. The tubes were centrifuged for 30 s at 14 000× *g*, and the supernatant was collected and concentrated to complete dryness. Metabolite profiling was performed using gas chromatography (GC) coupled to time‐of‐flight mass spectrometry (TOF‐MS). Restek corporation Rtx‐5Sil MS capillary column was used for metabolite separation.

### 2.5 Data acquisition and statistical analyses

The samples were derivatized for GC‐TOF‐MS analysis.^41^ Data values are represented as peak heights, comprising the quantification ion (m/z value) with the respective retention index, allowing for increased precision in identifying lower abundant metabolites. Raw reports contained deconvoluted mass spectra, retention indices, unique ions, standard compound identifiers, compound names, class annotations, and KEGG (version 103.0^42^) and PubChem (version 1.7.2 beta^43^) compound identifiers (CIDs). SMILES codes were generated using the PubChem Identifier Exchange Service. Univariate analysis involved independent sample t‐tests between each pair of groups to test for significantly different metabolites. False discovery rate (FDR) correction for the *p*‐values was used with a *p*‐value <.5 to indicate statistical significance. Log_2_FC line plots accounted for fold changes calculated by dividing the mean of the infected groups by that of the noninfected group. Line plots and partial least squares‐discriminant analysis (PLS‐DA) plots were generated using RStudio (version 4.1.2^44^). Statistical data, including fold changes and *p*‐values, were used to generate network maps using Metamapp (version 2.0.1^45^). An output file was saved as a Cytoscape (version 3.9.0^46^) node attribute file for significantly up or downregulated metabolites. ChemRICH, which employs Tanimoto substructure chemical similarity coefficients, was used to cluster metabolites into non‐overlapping chemical groups.^47^ Fold changes for ChemRICH and Metamapp input were calculated by dividing the median of infected groups by the median of noninfected groups.^48^ Statistically significant chemical classes were determined using ChemRICH by taking the p‐values and median fold changes.^47^ Downregulated and upregulated chemical groups were determined by taking the increased ratio originally on a 0 to +1 scale with 0.5 as the median and converting it to a −1 to +1 scale with 0 as the median.

## 3 RESULTS

### 3.1 HAdV‐5 infection resulted in distinct group clustering, while viral genes were expressed across all groups and times

HEK293 cells were used as the model cell line in this study, as previous research has uncovered their metabolic behavior.^49^, ^50^, ^51^ HEK293 aerobic glycolysis is similar to that in other commonly used cell lines.^51^ Concentration ratios of major carbon and energy metabolites in HEK293 cells, including glucose: lactate and glutamine: ammonia, have been well‐defined.^52^, ^53^ Aside from glycolytic and amino acid metabolite kinetics, however, understanding the lipid and nucleic acid profiles of the HEK293 cell line in response to human adenoviral infection is incomplete. Furthermore, HEK293 cells are susceptible to HAdV infection via the CAR protein.^54^ Compared to other non‐polarized cell lines, including A549 and HeLa, HEK293 cells express the highest amount of CAR^Ex8^ protein, a transmembrane isoform of CAR that mediates adenovirus entry.^55^ Furthermore, HEK293 susceptibility to HAdV infection was indicated by the highest expression of β‐galactosidase by the recombinant adenovirus.

To confirm viral infection, a subset of the treated samples was evaluated using RT‐qPCR. HAdV‐5 infection increased early‐phase gene expression, including E2A and E4, across time points and dosages, confirming viral infection (Figure S1A,B). 6HPI was the first time point in our analysis, and this was used as the baseline in the statistical analysis. The 0.5MOI mean relative fold changes for E2A were 1.15, 9.02, 5.75, and 5.31 across 6, 12, 24, and 36 h, respectively. The 1.0 MOI mean relative fold changes for E2A across time were 1.08, 227.53, 428.14, and 33.41, respectively. The 2.0 MOI mean fold changes for E2A across time were 1.06, 45.07, 244.55, and 129.46, respectively. The E4 mean relative fold changes for the 0.5MOI group over time were 1.07, 1.66, 5.25, and 4.29. 1.0MOI resulted in 1.17, 154.16, 476.70, and 26.55. 2.0MOI mean fold changes were 1.24, 40.45, 106.26, and 8.94. The evolution of infection groups by PLS‐DA revealed that noninfected cells clustered away from the infected cells across all treatments (Figure S2A–D).

### 3.2 HAdV‐5 infection across 36 h disrupts amino acid, lipid, nucleic acid, and sugar metabolism with notable downregulation

**FIGURE 1:**
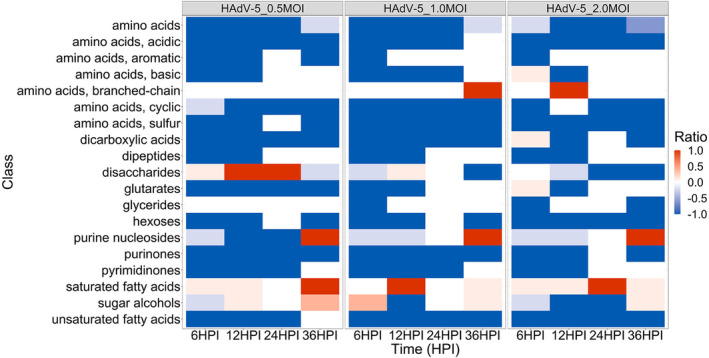
ChemRICH analysis reveals differential metabolite classes between infected and noninfected fibroblasts. Heatmap of altered metabolite classes. Metabolite classes are in rows across time, and the ratio of up‐ and downregulation corresponds to the color of each tile. Darker blue trends toward greater downregulation, while darker red trends toward greater upregulation.

After the viral infection was confirmed and responses were distinct between our control and treatment groups, we classified the directional changes of metabolites at each time point. A total of 182 individual metabolites were identified in our analysis, and all downstream metabolomic analyses were performed using these specific metabolites. Fold changes between noninfected cells and our three treatment groups were calculated. Using ChemRICH to evaluate statistical enrichment, we first evaluated the classes of metabolism that were enriched. Most enrichment was observed at 6 and 12HPI, indicated by the predominant downregulation of metabolic classes (Figure 1). We observed that most amino acids were immediately downregulated across all dosages. Metabolites involved in unsaturated fatty acid metabolism were also immediately downregulated. Nucleic acid metabolism was also affected in early infection, as indicated by the downregulation of purine nucleosides and purinones. Of note, additional aspects of metabolism were downregulated, including acidic amino acids, aromatic amino acids, dipeptides, and pyrimidine metabolism (Supporting Information Tables II–IV). Upregulation was observed in the levels of disaccharides, sugar alcohols, and saturated fatty acid metabolites. Using MetaMapp, we grouped the significantly altered metabolites based on biochemical and mass spectral similarities.^45^ Across all three dosages, our analyses revealed that HAdV‐5 infection disrupted multiple metabolic pathways in fibroblasts (Figures 1 and S3). We observed peak metabolic downregulation at 12HPI (Figure 1 and Supporting Information Tables V–VII). Across all three dosages, the number of significant metabolites detected at 24 HPI was reduced, suggesting a compensatory mechanism by the cells or the optimal effects of viral infection between 12 and 24 HPI. The data at 24HPI revealed fewer altered metabolic categories, with amino acid and unsaturated fatty acid classes being shared across all dosages (Figure 1 and Supporting Information Tables VIII–X). Network analyses revealed consistency in metabolic shifts across metabolite classes (Figure S5). Data at 36HPI revealed the upregulation of saturated fatty acids at an MOI of 0.5, and the downregulation of unsaturated fatty acid metabolism at 2.0 MOI (Figure 1). Across the three dosages, amino acid and dicarboxylic acid metabolism were downregulated (Supporting Information Tables XI–XIII). Network analyses of the perturbed metabolites revealed clustering of amino acids, carbohydrates, lipids, and nucleic acid metabolites (Figure S6).

### 3.3 HAdV‐5 infection induces a classical glycolytic interaction while perturbing cysteine, purine, and unsaturated fatty acid metabolism

**FIGURE 2:**
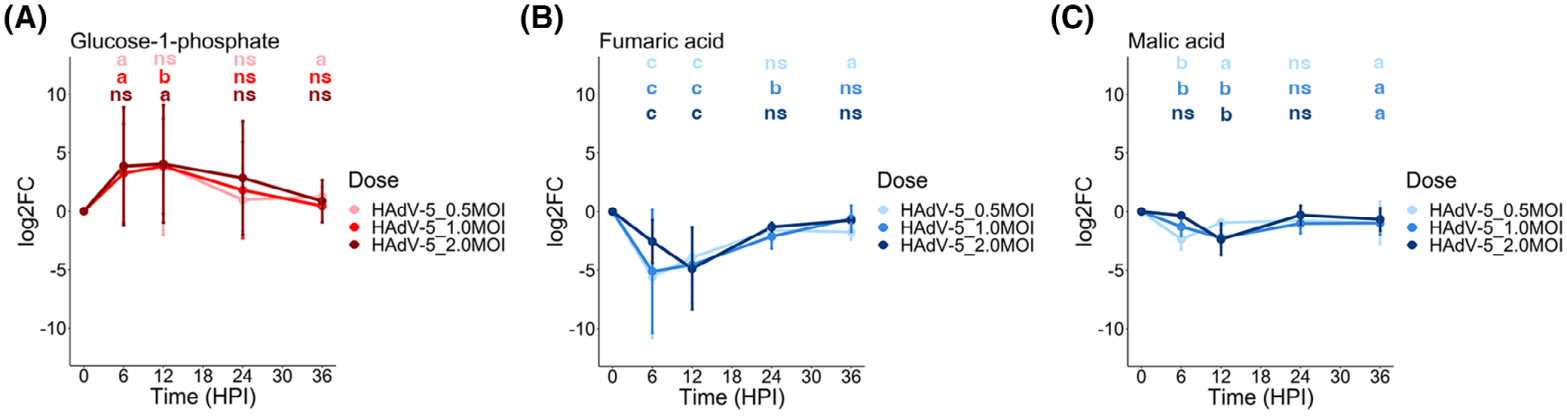
HAdV‐5 infection exhibits metabolic shifts consistent with classic glycolytic effects. (A) Line plot progression of glucose‐1‐phosphate log_2_‐fold change values relative to noninfected cells. (B) Line plot progression of fumaric acid log_2_‐fold change values relative to noninfected cells. (C) Line plot progression of malic acid log_2_‐fold change values relative to noninfected cells. Error bars indicate standard error from the mean (±SEM). False discovery rate (FDR) correction is used to calculate the *p*‐value. ^a^ *p* < .05, ^b^ *p* < .01, ^c^ *p* < .001, ns, not significant.

**FIGURE 3:**
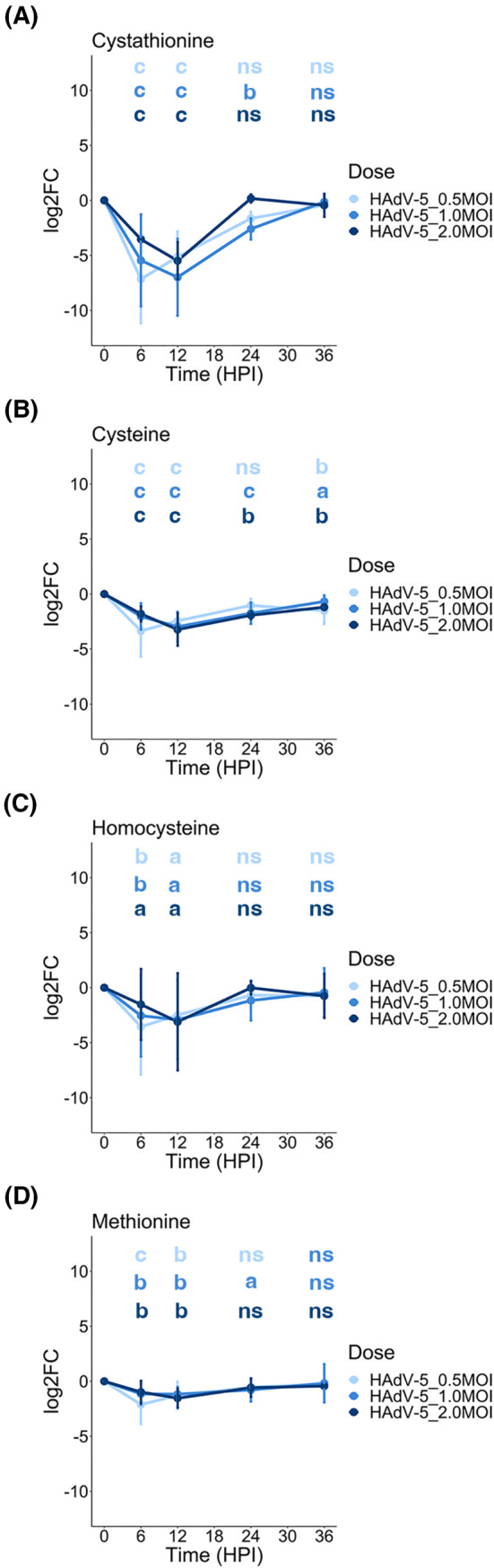
Cysteine metabolites exhibit significant downregulation, notably at 6 and 12 HPI. (A) Line plot progression of cystathionine log_2_‐fold change values relative to noninfected cells. (B) Line plot progression of cysteine log_2_‐fold change values relative to noninfected cells. (C) Line plot progression of homocysteine log_2_‐fold change values relative to noninfected cells. (D) Line plot progression of methionine log_2_‐fold change values relative to noninfected cells. Error bars indicate standard error from the mean (±SEM). False discovery rate (FDR) correction is used to calculate the *p*‐value. ^a^ *p* < .05, ^b^ *p* < .01, ^c^ *p* < .001, ns, not significant.

**FIGURE 4:**
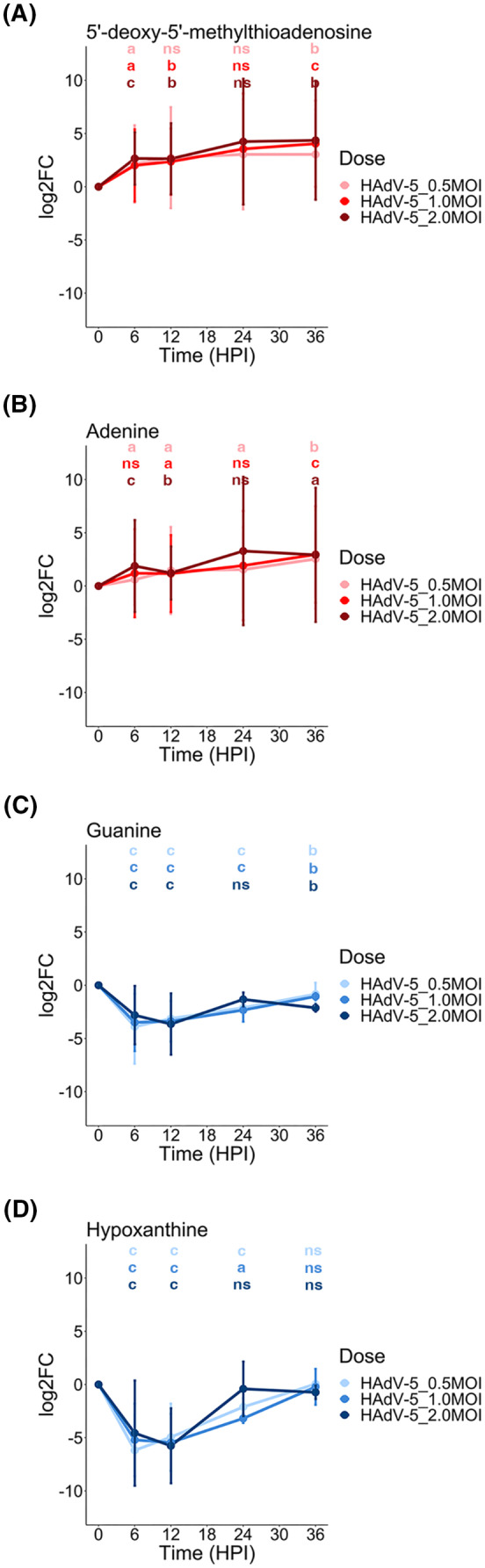
Purine metabolites exhibit dynamic changes across time. (A) Line plot progression of MTA log_2_‐fold change values relative to noninfected cells. (B) Line plot progression of adenine log_2_‐fold change values relative to noninfected cells. (C) Line plot progression of guanine log_2_‐fold change values relative to noninfected cells. (D) Line plot progression of hypoxanthine log_2_‐fold change values relative to noninfected cells. Error bars indicate standard error from the mean (±SEM). False discovery rate (FDR) correction is used to calculate the *p*‐value. ^a^ *p* < .05, ^b^ *p* < .01, ^c^ *p* < .001, ns, not significant.

**FIGURE 5:**
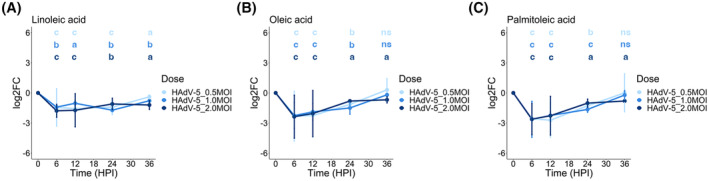
Unsaturated fatty acid metabolites exhibit significant downregulation, notably at 6 and 12 HPI prior to equilibrizing. (A) Line plot progression of linoleic acid log_2_‐fold change values relative to noninfected cells. (B) Line plot progression of oleic acid log_2_‐fold change values relative to noninfected cells. (C) Line plot progression of palmitoleic acid log_2_‐fold change values relative to noninfected cells. Error bars indicate standard error from the mean (±SEM). False discovery rate (FDR) correction is used to calculate the *p*‐value. ^a^ *p* < .05, ^b^ *p* < .01, ^c^ *p* < .001, ns, not significant.

Adenoviruses typically upregulate glucose metabolism through glycolytic pathways. At our early time points, upregulation of glucose‐1‐phosphate was observed, and the TCA cycle metabolites fumaric acid and malic acid were downregulated (Figure 2). This indicates a shift in favor of glycolysis, as opposed to downstream pathways, notably the TCA cycle. Additionally, cysteine, purine, and unsaturated fatty acid metabolism were also altered. Log_2_FC line plots of cysteine derivatives associated with *amino acid and sulfur* clusters in the ChemRICH plots revealed prominent downregulation, notably at 6 and 12 HPI, with recovery after 12 HPI (Figure 3). The metabolites included cystathionine, cysteine, homocysteine, and methionine. Similarly, purine derivatives were constrained to purine nucleoside and purinone clusters. Purine metabolites adenine and MTA were upregulated through to 36 HPI, while the purine metabolites guanine and hypoxanthine tended to be downregulated (Figure 4). We also tracked the unsaturated fatty acid metabolites and their fold change values over time (Figure 5). The fold change data and network maps demonstrated that linoleic acid, oleic acid, and palmitoleic acid were downregulated across the time of infection, notably at the 2.0MOI dose, suggesting that long‐chain fatty acids may be more susceptible to HAdV‐induced alterations in metabolism than medium‐ and short‐chain fatty acids.

## 4 DISCUSSION

The last decade has witnessed an explosion of studies examining viruses and their global cellular manipulation of host metabolism. The advent of analytical technologies has led to the discovery of new metabolites and their respective pathways that are used by multiple viral species. In the case of human adenoviruses, the mechanisms regulating glycolysis and cellular respiration upon adenoviral infection are now being elucidated.^56^, ^57^ While individual proteins of HAdV‐5 have been shown to disrupt cellular metabolism,^8^, ^32^, ^33^ a more comprehensive examination of the impacted host metabolic pathways in response to an intact adenovirus is lacking. Therefore, we sought to better characterize the intracellular metabolic response to HAdV‐5 infection in quiescent fibroblasts, HEK293 cells, using multiple dosages and durations of infection. HEK293 cells have previously been used to examine adenoviral disruption of host metabolism, although these studies used more targeted approaches.^34^, ^58^ In the present study, 182 metabolites were identified, with the majority centered on amino acids, carbohydrates, lipids, and nucleic acid metabolism. We discovered that the greatest metabolic response was as early as 6 HPI across all dosages, with peak metabolic dysregulation observed at 12 HPI, indicating how rapidly a viral infection can disrupt host metabolism independent of the infection dose. At 24 HPI, a marked reduction in the number of statistically altered metabolites was observed, which likely reflects the post‐peak depletion of metabolic substrates associated with the late‐phase genetic activation of the adenoviral life cycle. Because most adenoviral studies on the disruption of metabolism utilize a high viral titer at infection or a comparison of high and low doses on a 10‐fold scale, we purposefully examined the impact of infection on a narrower, more refined (two‐fold) scale. Interestingly, the profiles of the 0.5, 1.0, and 2.0 MOI‐treated groups were similar, suggesting that a minimum threshold to activate a host response can be achieved at a relatively low exposure and in a short period. Nonetheless, unique metabolic responses were observed across dosages and measured time intervals post‐infection.

Consistent with previous studies, we observed a typical upregulation of glycolysis, which can be inferred by the upregulation of glycolytic and downregulation of Krebs cycle metabolites. However, the most unique and significant contribution of the present study is the discovery of profound changes in the metabolism of cysteine, purine, and unsaturated fatty acids.

### 4.1 Cysteine metabolism

**FIGURE 6:**
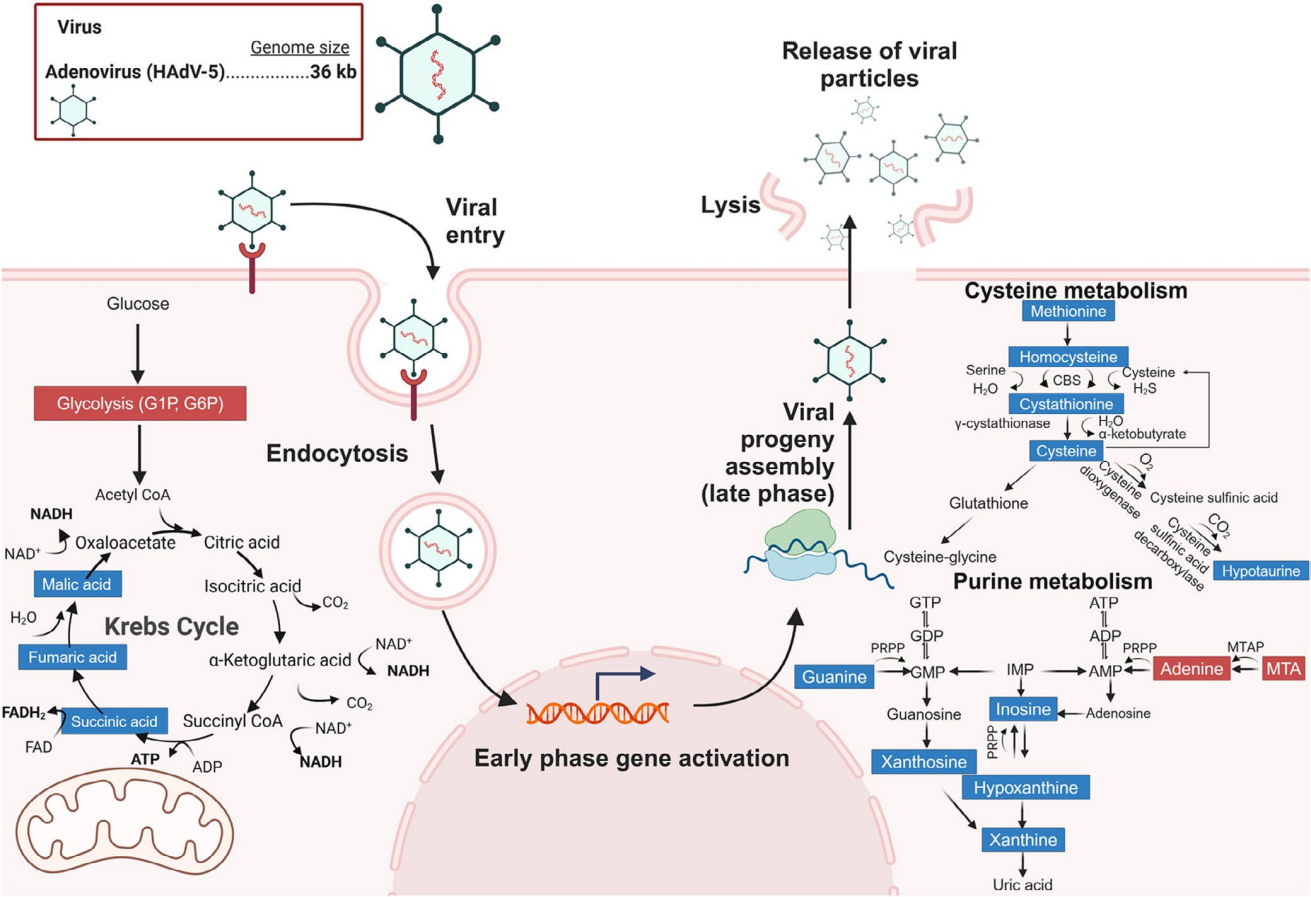
Pathways implicated in this study. Overview of HAdV‐5 life cycle, with perturbed pathways. Red labels indicate upregulation and blue labels indicate downregulation. Made with BioRender.com. ADP, adenosine diphosphate; AMP, adenosine monophosphate; ATP, Adenosine triphosphate; CBS, cystathionine‐β‐synthase; FAD, flavin adenine dinucleotide; FADH_2_, flavin adenine dinucleotide (reduced form); G1P, glucose‐1‐phosphate; G6P, glucose‐6‐phosphate; GDP, guanosine diphosphate; GMP, guanosine monophosphate; GTP, guanosine triphosphate; IMP, inosine monophosphate; MTA, 5′‐deoxy‐5′methylthioadenosine; MTAP, 5′‐deoxy‐5′methylthioadenosine phosphorylase; NAD^+^, nicotinamide adenine dinucleotide; NADH, nicotinamide adenine dinucleotide (reduced form); PRPP, phosphoribosyl pyrophosphate.

The most notable cysteine metabolites altered throughout the infection timeline were cysteine, cystathionine, homocysteine, hypotaurine, and methionine. In the presence of sufficient methionine, homocysteine is produced, which in turn forms cystathionine through cystathionine‐β‐synthase (CBS) and transsulfuration.^59^ CBS catalyzes the endogenous synthesis of cystathionine, in which serine undergoes condensation with homocysteine.^59^, ^60^ Cystathionine is then converted into cysteine by γ‐cystathionase. After its formation, cysteine can continue down two pathways. The first involves the formation of glutathione, which is the most prominent non‐enzymatic antioxidant stimulated during redox stress.^61^, ^62^, ^63^ Alternatively, cysteine can be converted into cysteine sulfinic acid, which subsequently is decarboxylated to hypotaurine^64^, ^65^, ^66^ (Figure 6).

Cystathionine has anti‐inflammatory and anti‐apoptotic potential, in addition to eliminating superoxide radicals.^59^ In our study, cystathionine was reduced in a dose‐dependent manner, suggesting that HAdV infection can compromise a cell's self‐defense against a viral insult by reducing its anti‐inflammatory and antioxidant potential. Additionally, homocysteine exhibited a similar trend to that of cystathionine. Homocysteine is a sulfhydryl‐containing, non‐proteinogenic amino acid essential for cell cycle progression and homeostasis.^64^ Dysregulation of homocysteine, including hypohomocysteinemia and hyperhomocysteinemia, has been linked to multiple diseases, rendering changes in homocysteine levels a useful marker for impaired amino acid metabolism depending on the condition.^67^, ^68^, ^69^ A meta‐analysis of HIV‐infected subjects under antiretroviral therapy revealed that mean homocysteine levels were elevated compared to non‐treated HIV subjects,^70^ suggesting that the maintenance of homocysteine levels is important for sustaining cellular health. Homocysteine has also been implicated as a potential biomarker of COVID‐19 infection as levels were increased in COVID‐19 patients^71^, ^72^ and remained elevated for 3 months in subjects diagnosed with COVID‐19‐associated pneumonia.^71^ While our results substantiate the idea that viral infections perturb homocysteine metabolism, resulting in profound changes in levels, they advance our knowledge by indicating that the initial insult may suppress cysteine metabolism. Furthermore, post‐infection elevation in homocysteine levels may serve as an indication of cellular rebound to support cell recovery. Future studies that include targeted examination of enzymes that regulate cysteine metabolism will provide additional insights into whether the changes are due to increased utilization/degradation and/or decreased de novo synthesis.

### 4.2 Purine metabolism

While serving as structural components of nucleic acids, adenine and guanine may also contribute to the regulation of cellular energetics and multiple signal transduction pathways.^73^ Cells require purines for their growth, proliferation, and survival. In addition to adenine and guanine, metabolites involved in purine metabolism were significantly altered in our study, including 5′‐deoxy‐5’‐methylthioadenosine (MTA), inosine, hypoxanthine, and xanthine. Nucleoside inosine is converted to hypoxanthine, which is then converted into xanthine.^74^, ^75^, ^76^, ^77^ Xanthine can also form after the conversion of guanosine into xanthosine. After xanthine synthesis, uric acid results from the oxidation of xanthine (Figure 6)^78^ and may possess antioxidant potential. In the present study, adenine and MTA were upregulated across most time points, while guanine, hypoxanthine, and xanthine were downregulated, notably at 6 and 12 HPI. This reciprocal change in adenine and xanthines suggests that ATP utilization is increased and adenine recycling is increased to support the increased energetic burden induced by viral infection. The reduction in xanthines suggests that the antioxidant potential of the host is compromised during adenoviral infection.

### 4.3 Unsaturated fatty acid metabolism

Unsaturated fatty acids can be divided into two categories: monounsaturated fatty acids (MUFA) and polyunsaturated fatty acids (PUFA). Monounsaturated fatty acids function in apoptosis, cell proliferation and growth, membrane dynamics, and the unfolded protein response.^79^ In the present study, oleic acid was downregulated across all time points. Oleic acid synthesis is achieved when the saturated FA, palmitic acid, is formed when acetyl CoA and malonyl CoA are converted by fatty acid synthase.^80^ Palmitic acid is then desaturated to palmitoleic acid or stearic acid, another saturated fatty acid. Stearic acid is desaturated to form oleic acid. Palmitoleic acid was downregulated, notably at 6 and 12 HPI. In addition to MUFAs, PUFAs were also downregulated, most notably linoleic acid (18:2).^81^ Alpha‐linoleic acid's (αLA) antiviral potential against multiple viral species, including Dengue, SARS‐CoV‐2, and Zika, has been reported. Alpha‐LA inhibited Zika mRNA production in cells without loss of cell viability while also interrupting binding and cellular entry of the virus.^82^ Therefore, our data on HAdV‐induced downregulation of PUFAs suggest that the persistence of a viral infection may be perpetuated by the immediate suppression of key PUFAs during the initial phase. Alternatively, the initial rapid decrease in MUFAs and PUFAs may reflect a rapid shift in increased lipid metabolism to support the increased energetic burden induced by an initial viral insult. Regardless of the purpose, the data suggest that dietary supplements of PUFAs may help alleviate the cellular energetic burden and decrease the recovery time.

### 4.4 Summary

Our results revealed unique metabolic pathways perturbed by adenoviral infection. Given that viral infections are commonly associated with primary disruption of the lung epithelium, our results provide insights into additional sites of cellular metabolism. The data demonstrated the downregulation of TCA cycle intermediates and upregulation of glycolytic intermediates, with simultaneous changes in cysteine, purine, and unsaturated fatty acid metabolism. Changes in these metabolic pathways are indicative of an increase in glucose metabolism, likely supporting the rapid increase in cellular energetics induced by a viral insult. The infected cells also showed increased purine (adenine) recycling, which may support the increased energetic burden at the expense of producing uric acid, which may help ameliorate the potential for inflammatory and oxidative injury. The data also suggest that adenoviral infection induces a shift in cellular priority toward supporting the energetic burden at the expense of increasing the cell's susceptibility to inflammatory and oxidative injury during the initial and early phases of the infection that persist over the first 24 h. This is supported by the downregulation of unsaturated fatty acid metabolites and a vast number of amino acid metabolites, particularly cysteine pathway intermediates. The doses utilized here demonstrate that adenoviral infections at relatively low doses are sufficient to induce profound effects on host metabolism and help identify a minimal effective dose to reach a metabolic threshold response. The time points used revealed that the response threshold was achieved relatively rapidly, which suggests that more immediate intervention should help ameliorate the duration and impact of the infection and reduce recovery time.

### 4.5 Limitations and conclusion

While there is value in exploring HAdV‐5 infection in HEK293 cells, it is important to note that HEK293 cells are transformed with HAdV E1A and E1B genes to facilitate immortalization and aid in the production of recombinant viruses during vaccine development.^83^ Nonetheless, insights into HEK293 metabolism upon HAdV infection have been shown in previous studies, including the present study.^34^ To capture in vitro metabolome changes in response to HAdV infection that would better model natural infection consequences, it would be sensible to apply a similar platform to the one in the present study in a lung epithelial cell line and/or gastrointestinal cell line as these are natural tissue targets of HAdVs, including HAdV‐5.^17^, ^84^ To ensure an adequate return of the sample data, the experiments were conducted in sextuplicate. Of note, one replicate at 12HPI from the 2.0MOI group overlapped with the noninfected cluster in the PLS‐DA, signifying an outlying sample, which was removed from the downstream analyses. Furthermore, one replicate from the 0.5MOI group at 36HPI failed the injection and was not included in the downstream analyses. Nonetheless, subtle differences were observed in the metabolome profiles across doses. In summary, we envision that these findings will facilitate the implementation of more mechanistic studies to assist in the development of more robust therapeutic interventions (pharmaceutical, dietary, or otherwise) to combat adenoviral infections.

## AUTHOR CONTRIBUTIONS

Bailey‐J C. Sanchez, Rudy M. Ortiz, and Juris A. Grasis conceived and designed the experiments. Bailey‐J C. Sanchez conducted the experiments and analyzed the data. Rudy M. Ortiz and Juris A. Grasis interpreted the data and provided feedback on the results. Bailey‐J C. Sanchez wrote the first draft of the manuscript, including figures. Rudy M. Ortiz and Juris A. Grasis provided input on the manuscript and figures. Juris A. Grasis provided revisions to the manuscript and figures. Rudy M. Ortiz and Juris A. Grasis provided material support for conducting the experiments.

## FUNDING INFORMATION

This research was supported by NSF BII: Host‐Virus Evolutionary Dynamics Institute (JAG, 2119968). BJS was supported by a fellowship from the USDA HSI Education Training grant (2021‐03397). This work was supported by NIH grant U2C‐DK119886 and OT2‐OD030544 grants.

## DISCLOSURES

The authors declare no conflicts of interest that could be perceived to bias their work, and all funding sources have been disclosed.

## Supporting information


Data S1.

Data S2.

## Data Availability

The raw metabolomics data and code for statistical generation are deposited in the Dryad data repository (DOI:10.5061/dryad.z612jm6n2). This study is also available at the NIH Common Fund's National Metabolomics Data Repository (NMDR), the Metabolomics Workbench,^85^ and has been assigned Study ID ST003649. The data can be accessed directly via its Project DOI: https://doi.org/10.21228/M8MV7R.
