# Supplementary material for: Human adenovirus serotype 5 infection dysregulates cysteine, purine, and unsaturated fatty acid metabolism in fibroblasts
Source: FASEB J. 2025 Mar 7;39(5):e70411. doi: 10.1096/fj.202402726R (PMC11887610; doi:10.1096/fj.202402726R)
Supplement: Supplementary file 1 — Data S1. [file FSB2-39-e70411-s002.pdf]

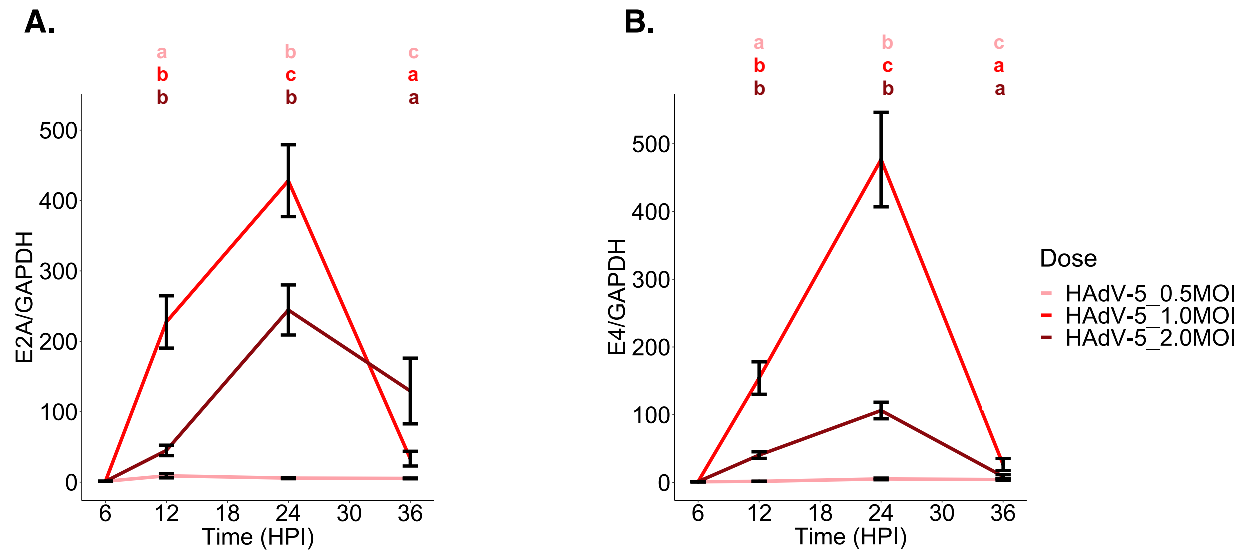

Supplemental Figure 1: **RT-qPCR data confirms viral infection at all time points in this study**. **A.** Relative. gene expression results for the Pan E2A gene for 0.5MOI, 1.0MOI, and 2.0MOI dosages across time. **B.** Relative gene expression results for Pan E4 gene for 0.5MOI, 1.0MOI, and 2.0MOI dosages across time. The 6HPI group was used for comparison across all experiments. Experiments were performed in sextuplicate. Data shown as standard error from the mean (±SEM), and statistical analysis was performed using the two-group t-test. **a** = $P < 0.05$, **b** = $P < 0.01$, **c** = $P < 0.001$, **ns** = not significant.

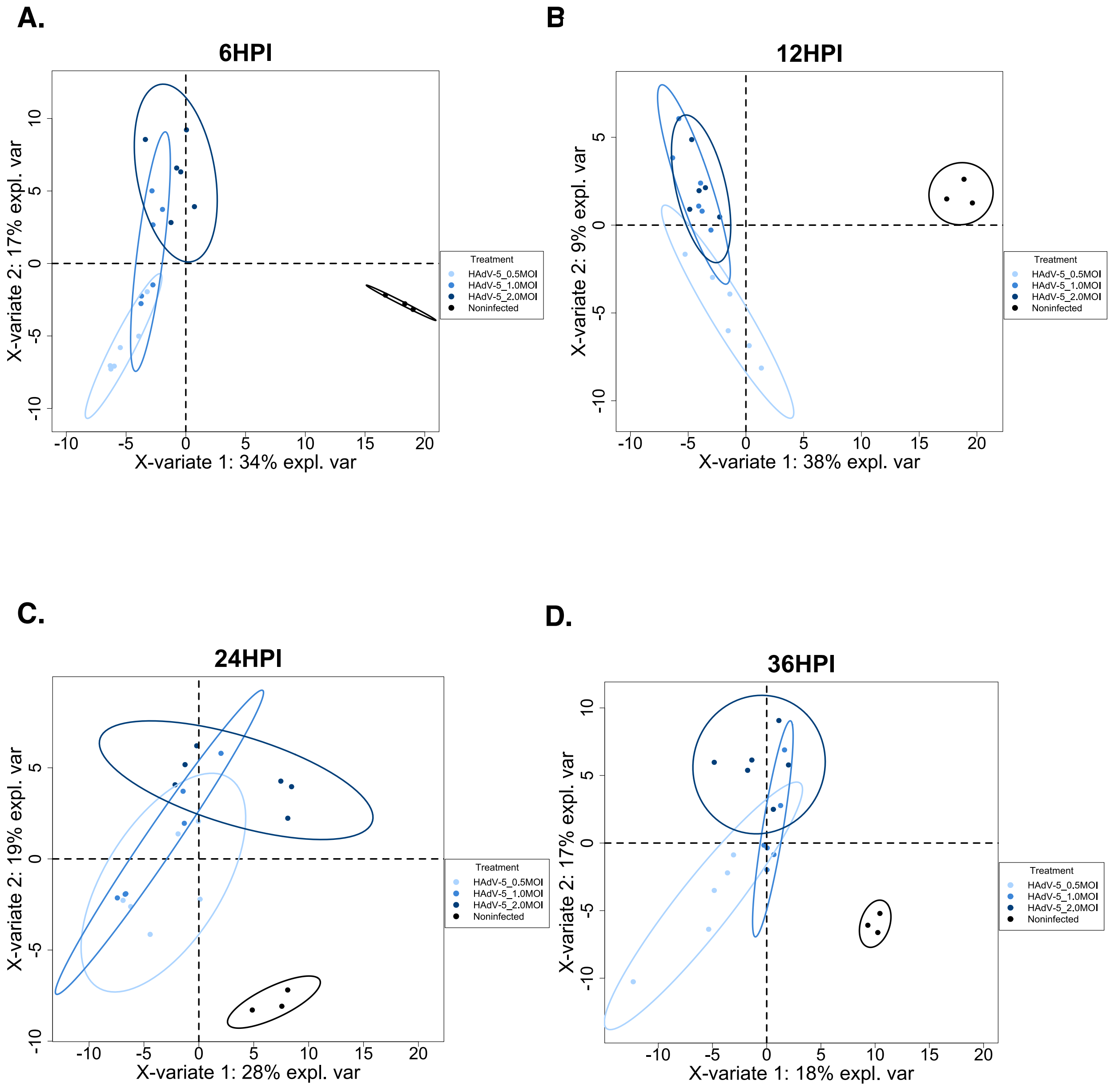

Supplemental Figure 2: **Partial least squares-discriminant analysis (PLS-DA) reveals separation between control and experimental conditions**. **A.** PLS-DA for 6HPI comparisons. **B.** PLS-DA for 12HPI comparisons. **C.** PLS-DA for 24 HPI comparisons. **D.** PLS-DA comparison for 36HPI comparisons.

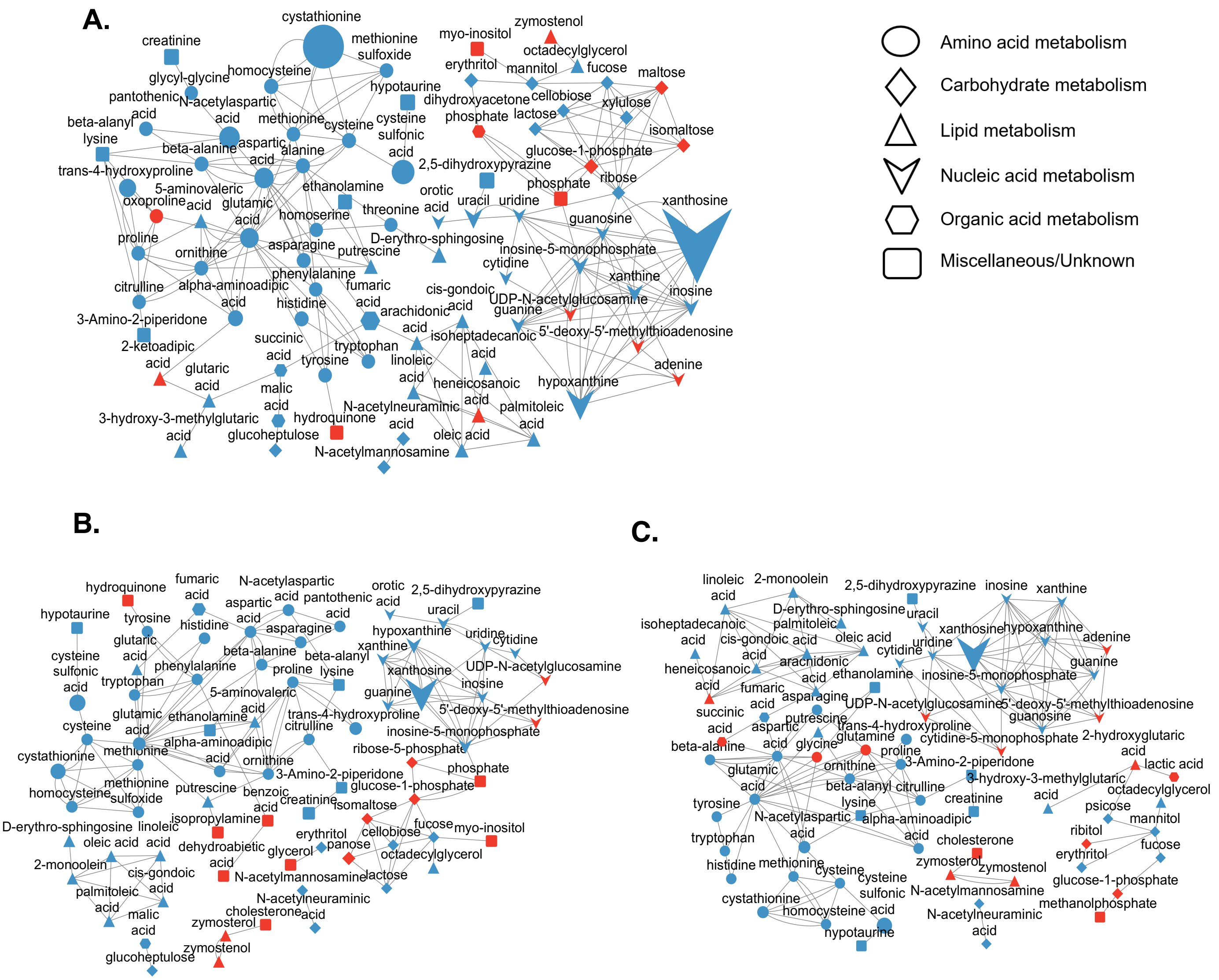

Supplemental Figure 3: Network analyses at 6 HPI show early perturbation of individual metabolites across multiple categories. **A.** Network map analyzing biochemical relationship between metabolites significantly altered in the 0.5MOI/noninfected comparison. **B.** Network map analyzing biochemical relationship between metabolites significantly altered in the 1.0MOI/noninfected comparison. **C.** Network map analyzing biochemical relationship between metabolites significantly altered in the 2.0MOI/noninfected comparison. The size of each shape corresponds to the fold change value calculated in MetaMapp. Blue shapes represent downregulated metabolites, while red shapes indicate upregulated metabolites.

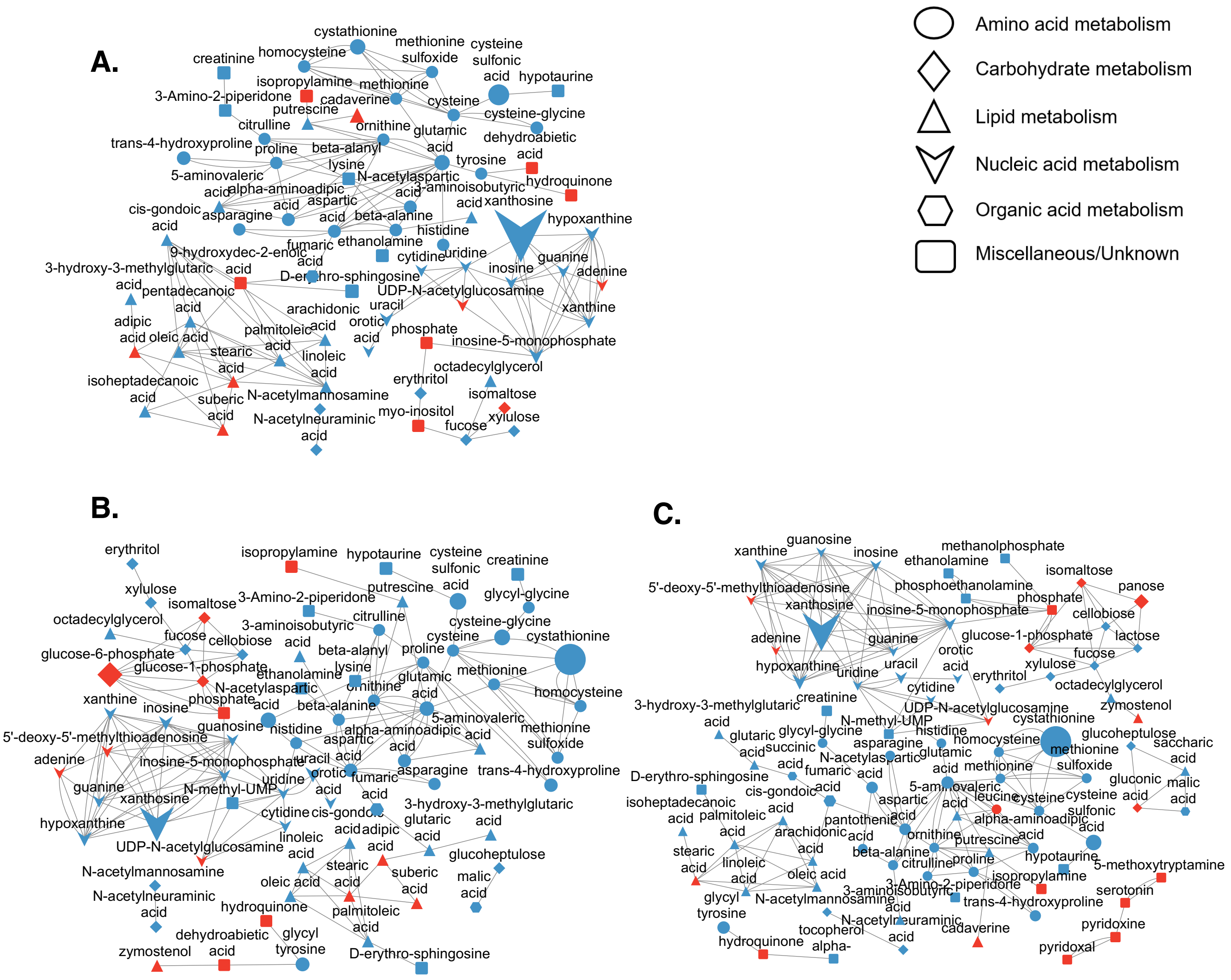

Supplemental Figure 4: Network analyses reveal continued metabolic perturbations at 12 HPI. A. Network map analyzing biochemical relationship between metabolites significantly altered in the 0.5MOI/noninfected comparison. **B.** Network map analyzing biochemical relationship between metabolites significantly altered in the 1.0MOI/noninfected comparison. **C.** Network map analyzing biochemical relationship between metabolites significantly altered in the 2.0MOI/noninfected comparison. The size of each shape corresponds to the fold change value calculated in MetaMapp. Blue shapes represent downregulated metabolites, while red shapes indicate upregulated metabolites.

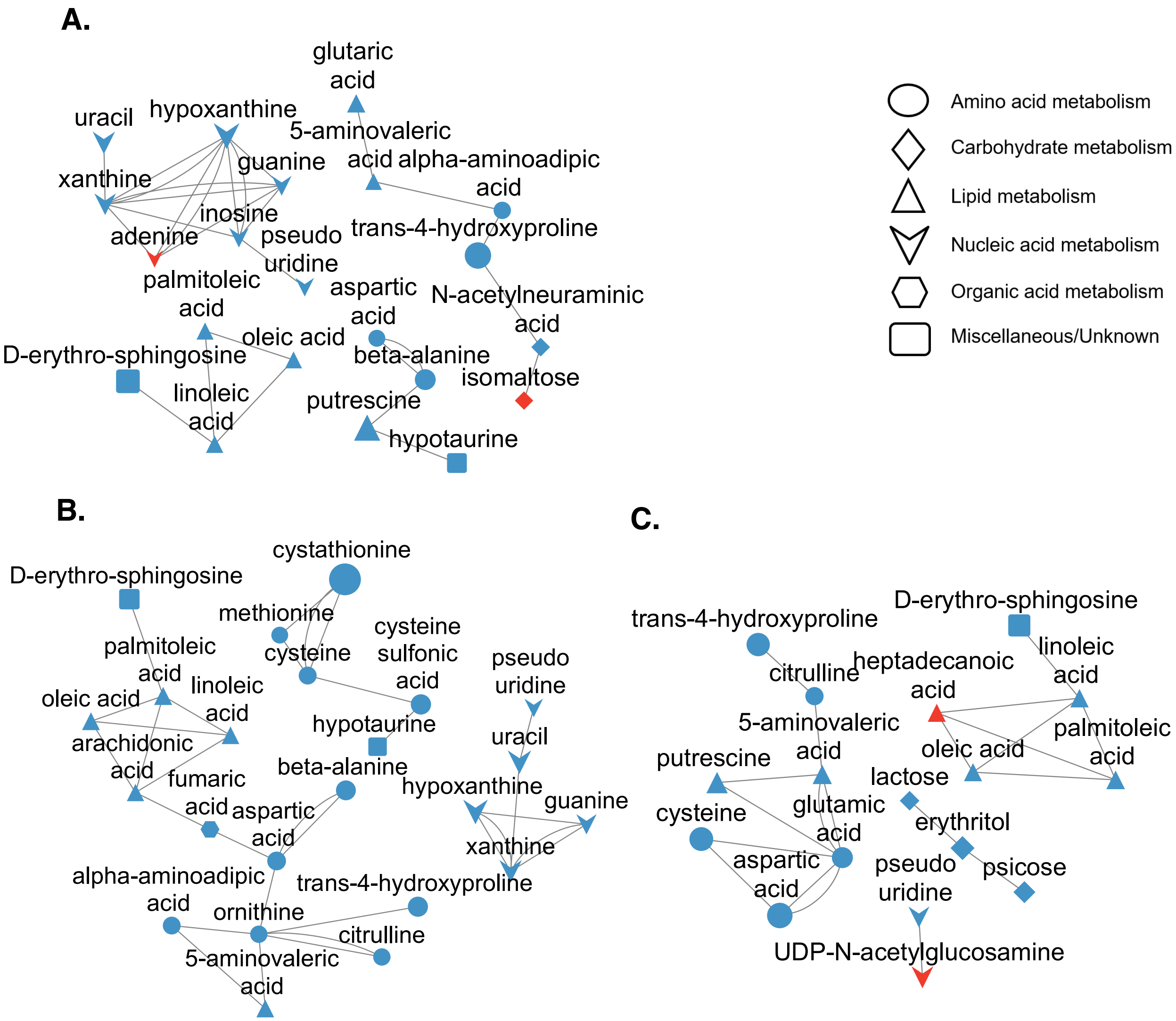

Supplemental Figure 5: **Network analyses at 24HPI reveal marked reduction in metabolite involvement across amino acid, lipid, and nucleic acid metabolism**. **A.** Network map analyzing biochemical relationship between metabolites significantly altered in the 0.5MOI/noninfected comparison. **B.** Network map analyzing biochemical relationship between metabolites significantly altered in the 1.0MOI/noninfected comparison. **C.** Network map analyzing biochemical relationship between metabolites significantly altered in the 2.0MOI/noninfected comparison. The size of each shape corresponds to the fold change value calculated in MetaMapp. Blue shapes represent downregulated metabolites, while red shapes indicate upregulated metabolites.

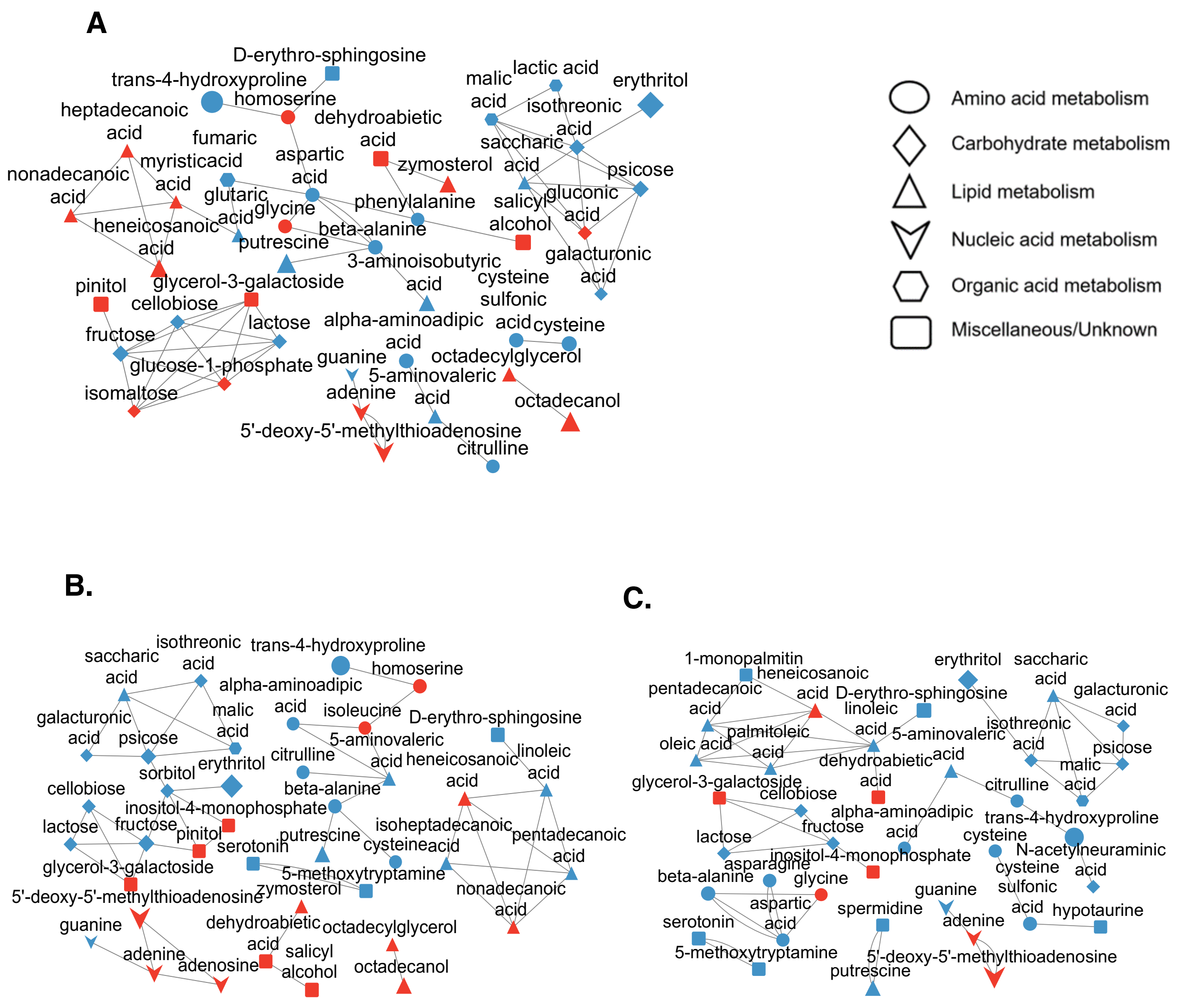

Supplemental Figure 6: **Network analyses at 36HPI yield increases in metabolite shifting across treated/untreated comparisons**. **A.** Network map analyzing biochemical relationship between metabolites significantly altered in the 0.5MOI/noninfected comparison. **B.** Network map analyzing biochemical relationship between metabolites significantly altered in the 1.0MOI/noninfected comparison. **C.** Network map analyzing biochemical relationship between metabolites significantly altered in the 2.0MOI/noninfected comparison. The size of each shape corresponds to the fold change value calculated in MetaMapp. Blue shapes represent downregulated metabolites, while red shapes indicate upregulated metabolites.
