# Supplementary material for: Human adenovirus serotype 5 infection dysregulates cysteine, purine, and unsaturated fatty acid metabolism in fibroblasts
Source: FASEB J. 2025 Mar 7;39(5):e70411. doi: 10.1096/fj.202402726R (PMC11887610; doi:10.1096/fj.202402726R)
Supplement: Supplementary file 2 — Data S2. [file FSB2-39-e70411-s001.pdf]

Supplemental Table I: Primers used in RT-qPCR analysis

Gene name	Gene ID	Primer sequence (5'-3')	Template location
GAPDH	2597	FOR:5'- CCAGCCGAGCCACATCGCTC-3'	50
		REV:5'-ATGAGCCCCAGCCTTCTCCAT-3'	409
Pan E1A	2659820	FOR:5'-CCGACACCGGGACTGAAAAT-3'	541
		REV:5'-AGCTGGTCCAAAAGACTGGC-3'	626
Pan E2A	5739976	FOR:5'-CGGTCTGGGCGTTAGGATAC-3'	22866
		REV:5'-TGCAGATCTCCAACACCGAC-3'	23036
Pan E4	2652989	FOR:5'-ACACGGCACCAGCTCAATC-3'	35600
		REV:5'-ATATACTCGCTCTGCACTTGGC-3'	35720

Supplemental Table II: ChemRICH cluster information for 0.5MOI_6HPI.

Cluster name	Cluster size	P-value	Transformed P-value	Altered metabolites	Up/down ratio
adipates	3	5.00E-03	9.99E+00	2	0.1
amino acids	9	4.60E-05	2.87E+01	6	-1
amino acids, acidic	3	3.60E-13	2.07E+01	3	-1
amino acids, aromatic	3	1.00E-09	0.00E+00	3	-1
amino acids, basic	3	1.00E+00	0.00E+00	1	-1
amino acids, branched-chain	3	1.00E+00	2.85E+01	0	0
amino acids, cyclic	4	4.40E-13	2.21E+01	4	-0.8
amino acids, sulfur	5	2.60E-10	0.00E+00	5	-1
benzene derivatives	3	1.00E+00	0.00E+00	0	0
biogenic polyamines	4	1.00E+00	0.00E+00	1	-1
cholestenes	4	1.00E+00	4.20E+00	1	1
dicarboxylic acids	5	1.50E-02	1.01E+01	3	-1
dipeptides	5	4.30E-05	7.90E+00	2	-1
disaccharides	6	3.70E-04	0.00E+00	4	0.1
ethanolamines	3	1.00E+00	2.98E+00	1	-1
glutarates	3	5.10E-02	0.00E+00	2	-1
glyceric acids	3	1.00E+00	0.00E+00	0	0
glycerides	3	1.00E+00	1.21E+01	0	0
hexosephosphates	6	5.80E-06	0.00E+00	2	0.1
hexoses	3	1.00E+00	3.12E+00	1	-1
pentoses	3	4.40E-02	7.31E+00	2	-1

purine nucleosides	7	6.70E-04	1.53E+01	5	-0.8
purinones	3	2.20E-07	3.24E+00	3	-1
pyrimidine nucleosides	3	3.90E-02	3.30E+00	2	-1
pyrimidinones	3	3.70E-02	7.26E+00	2	-1
saturated fatty acids	14	7.00E-04	2.96E+00	2	0.1
sugar alcohols	9	5.20E-02	9.85E+00	3	-0.6
unsaturated fatty acids	6	5.30E-05	9.99E+00	5	-1

Supplemental Table III: ChemRICH cluster information for 1.0MOI_6HPI.

Cluster name	Cluster size	P-value	Transformed P-value	Altered metabolites	Up/down ratio
adipates	3	1.00E+00	0.00E+00	1	-1
amino acids	8	1.30E-02	4.34E+00	3	-1
amino acids, acidic	3	3.10E-14	3.11E+01	3	-1
amino acids, aromatic	3	1.00E-06	1.38E+01	3	-1
amino acids, basic	3	1.00E+00	0.00E+00	1	-1
amino acids, branched-chain	3	1.00E+00	0.00E+00	0	0
amino acids, cyclic	4	4.00E-03	5.52E+00	3	-1
amino acids, sulfur	4	2.60E-10	2.21E+01	4	-1
benzene derivatives	3	1.00E+00	0.00E+00	1	1
biogenic polyamines	4	1.00E+00	0.00E+00	1	-1
cholestenes	4	8.20E-03	4.80E+00	3	1
dicarboxylic acids	5	1.60E-01	1.83E+00	2	-1
dipeptides	5	1.00E+00	0.00E+00	1	-1
disaccharides	6	9.50E-03	4.66E+00	3	-0.6
ethanolamines	3	1.00E+00	0.00E+00	1	-1
glutarates	3	1.00E+00	0.00E+00	1	-1
glyceric acids	3	1.00E+00	0.00E+00	0	0
glycerides	3	1.00E+00	0.00E+00	1	-1
hexosephosphates	6	8.20E-06	1.17E+01	3	0.4
hexoses	3	1.00E+00	0.00E+00	1	-1
pentoses	3	1.00E+00	0.00E+00	0	0

purine nucleosides	7	1.30E-02	4.34E+00	4	-0.8
purinones	3	2.20E-07	1.53E+01	3	-1
pyrimidine nucleosides	3	3.90E-02	3.24E+00	2	-1
pyrimidinones	3	3.70E-02	3.30E+00	2	-1
saturated fatty acids	14	1.00E+00	0.00E+00	0	0
sugar alcohols	9	1.40E-02	4.27E+00	3	0.4
unsaturated fatty acids	6	2.00E-03	6.21E+00	4	-1

Supplemental Table IV: ChemRICH cluster information for 2.0MOI_6HPI.

Cluster name	Cluster size	P-value	Transformed P-value	Altered metabolites	Up/down ratio
adipates	3	1.00E+00	0.00E+00	1	-1
amino acids	9	2.70E-02	3.61E+00	4	-0.6
amino acids, acidic	3	4.40E-11	2.38E+01	3	-1
amino acids, aromatic	3	4.10E-02	3.19E+00	2	-1
amino acids, basic	3	6.40E-04	7.35E+00	2	0.1
amino acids, branched-chain	3	1.00E+00	0.00E+00	0	0
amino acids, cyclic	4	4.00E-03	5.52E+00	3	-1
amino acids, sulfur	5	9.80E-06	1.15E+01	4	-1
benzene derivatives	3	1.00E+00	0.00E+00	0	0
biogenic polyamines	4	1.00E+00	0.00E+00	1	-1
cholestenes	4	6.10E-03	5.10E+00	3	1
dicarboxylic acids	5	9.50E-02	2.35E+00	2	0.1
dipeptides	5	1.00E+00	0.00E+00	1	-1
disaccharides	6	1.00E+00	0.00E+00	0	0
ethanolamines	3	1.00E+00	0.00E+00	1	-1
glutarates	3	5.20E-02	2.96E+00	2	0.1
glyceric acids	3	1.00E+00	0.00E+00	0	0
glycerides	3	1.00E+00	0.00E+00	1	-1
hexosephosphates	6	1.10E-03	6.81E+00	2	0.1
hexoses	3	3.90E-02	3.24E+00	2	-1
pentoses	3	1.00E+00	0.00E+00	0	0

purine nucleosides	7	1.60E-05	1.10E+01	5	-0.8
purinones	3	2.20E-07	1.53E+01	3	-1
pyrimidine nucleosides	3	3.90E-02	3.24E+00	2	-1
pyrimidinones	3	1.00E+00	0.00E+00	1	-1
saturated fatty acids	14	1.20E-02	4.42E+00	2	0.1
sugar alcohols	9	6.80E-03	4.99E+00	3	-0.6
unsaturated fatty acids	6	5.30E-05	9.85E+00	5	-1

Supplemental Table V: ChemRICH cluster information for 0.5MOI_12HPI.

Cluster name	Cluster size	P-value	Transformed P-value	Altered metabolites	Up/down ratio
adipates	3	8.00E-04	4.34E+00	5	0.1
amino acids	9	1.30E-02	2.07E+01	3	-1
amino acids, acidic	3	1.00E-09	0.00E+00	1	-1
amino acids, aromatic	3	1.00E+00	0.00E+00	1	-1
amino acids, basic	3	1.00E+00	0.00E+00	0	-1
amino acids, branched-chain	3	1.00E+00	6.12E+00	3	0
amino acids, cyclic	4	2.20E-03	1.77E+01	5	-1
amino acids, sulfur	5	2.10E-08	0.00E+00	0	-1
benzene derivatives	3	1.00E+00	2.21E+00	2	0
biogenic polyamines	4	1.10E-01	0.00E+00	0	0.1
cholestenes	4	1.00E+00	0.00E+00	1	0
dicarboxylic acids	5	1.00E+00	4.92E+00	2	-1
dipeptides	5	7.30E-03	0.00E+00	1	-1
disaccharides	6	1.00E+00	0.00E+00	1	1
ethanolamines	3	1.00E+00	0.00E+00	1	-1
glutarates	3	1.00E+00	0.00E+00	0	-1
glyceric acids	3	1.00E+00	0.00E+00	0	0
glycerides	3	1.00E+00	0.00E+00	1	0
hexosephosphates	6	1.00E+00	0.00E+00	1	-1
hexoses	3	1.00E+00	0.00E+00	1	-1
pentoses	3	1.00E+00	2.78E+00	3	-1

purine nucleosides	7	6.20E-02	1.33E+01	3	-1
purinones	3	1.70E-06	3.22E+00	2	-1
pyrimidine nucleosides	3	4.00E-02	2.94E+00	2	-1
pyrimidinones	3	5.30E-02	5.20E+00	4	-1
saturated fatty acids	14	5.50E-03	9.94E-01	2	0.1
sugar alcohols	9	3.70E-01	1.04E+01	5	0.1
unsaturated fatty acids	6	3.10E-05	4.34E+00	5	-1

Supplemental Table VI: ChemRICH cluster information for 1.0MOI_12HPI.

Cluster name	Cluster size	P-value	Transformed P-value	Altered metabolites	Up/down ratio
adipates	3	2.90E-03	5.84E+00	5	-1
amino acids	9	7.80E-16	3.48E+01	3	-1
amino acids, acidic	3	1.00E+00	0.00E+00	0	0
amino acids, aromatic	3	1.00E+00	0.00E+00	1	-1
amino acids, basic	3	1.00E+00	0.00E+00	0	0
amino acids, branched-chain	3	2.20E-03	6.12E+00	3	-1
amino acids, cyclic	4	6.60E-09	1.88E+01	5	-1
amino acids, sulfur	5	1.00E+00	0.00E+00	0	0
benzene derivatives	3	1.00E+00	0.00E+00	1	-1
biogenic polyamines	4	1.00E+00	0.00E+00	1	1
cholestenes	4	7.00E-02	2.66E+00	2	-1
dicarboxylic acids	5	5.60E-05	9.79E+00	4	-1
dipeptides	5	6.80E-04	7.29E+00	2	0.1
disaccharides	6	1.00E+00	0.00E+00	1	-1
ethanolamines	3	3.70E-02	3.30E+00	2	-1
glutarates	3	1.00E+00	0.00E+00	0	0
glyceric acids	3	1.00E+00	0.00E+00	0	0
glycerides	3	1.00E-02	4.61E+00	3	0.4
hexosephosphates	6	1.00E+00	0.00E+00	1	-1
hexoses	3	1.00E+00	0.00E+00	1	-1
pentoses	3	5.80E-04	7.45E+00	5	-0.6

purine nucleosides	7	1.70E-06	1.33E+01	3	-1
purinones	3	4.40E-04	7.73E+00	2	-1
pyrimidine nucleosides	3	3.80E-02	3.27E+00	2	-1
pyrimidinones	3	4.20E-02	3.17E+00	2	1
saturated fatty acids	14	1.00E+00	0.00E+00	1	-1
sugar alcohols	9	2.20E-04	8.42E+00	4	-1
unsaturated fatty acids	6	2.90E-03	5.84E+00	5	-1

Supplemental Table VII: ChemRICH cluster information for 2.0MOI_12HPI.

Cluster name	Cluster size	P-value	Transformed P-value	Altered metabolites	Up/down ratio
adipates	3	1.00E+00	0.00E+00	1	-1
amino acids	9	3.40E-03	5.68E+00	5	-1
amino acids, acidic	3	6.60E-15	3.27E+01	3	-1
amino acids, aromatic	3	1.00E+00	0.00E+00	0	0
amino acids, basic	3	1.00E+00	0.00E+00	1	-1
amino acids, branched-chain	3	1.00E+00	0.00E+00	1	1
amino acids, cyclic	4	1.50E-04	8.80E+00	3	-1
amino acids, sulfur	4	1.00E-08	1.84E+01	4	-1
benzene derivatives	3	1.00E+00	0.00E+00	0	0
biogenic polyamines	4	1.00E-01	2.30E+00	2	0.1
cholestenes	4	1.00E+00	0.00E+00	1	1
dicarboxylic acids	5	8.40E-03	4.78E+00	3	-1
dipeptides	5	1.50E-05	1.11E+01	2	-1
disaccharides	6	8.60E-03	4.76E+00	3	-0.4
ethanolamines	3	2.50E-04	8.29E+00	2	-1
glutarates	3	2.20E-02	3.82E+00	2	-1
glyceric acids	3	1.00E+00	0.00E+00	0	0
glycerides	3	1.00E+00	0.00E+00	0	0
hexosephosphates	6	2.50E-03	5.99E+00	2	0.1
hexoses	3	1.00E+00	0.00E+00	1	-1
pentoses	3	1.00E+00	0.00E+00	1	-1

purine nucleosides	7	5.70E-04	7.47E+00	5	-0.6
purinones	3	2.70E-06	1.28E+01	3	-1
pyrimidine nucleosides	3	3.90E-02	3.24E+00	2	-1
pyrimidinones	3	3.90E-02	3.24E+00	2	-1
saturated fatty acids	14	1.10E-02	4.51E+00	2	0.1
sugar alcohols	9	1.00E+00	0.00E+00	1	-1
unsaturated fatty acids	6	3.80E-05	1.02E+01	5	-1

Supplemental Table VIII: ChemRICH cluster information for 0.5MOI_24HPI.

Cluster name	Cluster size	P-value	Transformed P-value	Altered metabolites	Up/down ratio
amino acids	8	1.50E-01	1.90E+00	2	-1
amino acids, acidic	3	1.00E+00	0.00E+00	1	-1
amino acids, aromatic	3	1.00E+00	0.00E+00	0	0
amino acids, basic	3	1.00E+00	0.00E+00	0	0
amino acids, branched-chain	3	1.00E+00	0.00E+00	0	0
amino acids, cyclic	3	1.00E+00	0.00E+00	1	-1
amino acids, sulfur	5	1.00E+00	0.00E+00	0	0
dicarboxylic acids	7	1.00E+00	0.00E+00	1	-1
dipeptides	3	1.00E+00	0.00E+00	0	0
disaccharides	5	1.00E+00	0.00E+00	1	1
glutarates	3	1.00E+00	0.00E+00	1	-1
glycerides	3	1.00E+00	0.00E+00	0	0
hexoses	3	1.00E+00	0.00E+00	0	0
purine nucleosides	6	1.00E+00	0.00E+00	1	-1
purinones	3	6.40E-05	9.66E+00	3	-1
pyrimidinones	3	1.00E+00	0.00E+00	1	-1
saturated fatty acids	9	1.00E+00	0.00E+00	0	0
sugar acids	3	1.00E+00	0.00E+00	0	0
sugar alcohols	7	1.00E+00	0.00E+00	0	0

unsaturated fatty acids	4	1.90E-03	6.27E+00	3	-1

Supplemental Table IX: ChemRICH cluster information for 1.0MOI_24HPI.

Cluster name	Cluster size	P-value	Transformed P-value	Altered metabolites	Up/down ratio
amino acids	8	7.70E-02	-2.56E+00	3	-1
amino acids, acidic	3	1.00E+00	0.00E+00	1	-1
amino acids, aromatic	3	1.00E+00	0.00E+00	0	0
amino acids, basic	3	1.00E+00	0.00E+00	1	-1
amino acids, branched-chain	3	1.00E+00	0.00E+00	0	0
amino acids, cyclic	3	1.00E+00	0.00E+00	1	-1
amino acids, sulfur	5	5.20E-03	-5.26E+00	4	-1
dicarboxylic acids	7	1.90E-01	-1.66E+00	2	-1
dipeptides	3	1.00E+00	0.00E+00	0	0
disaccharides	5	1.00E+00	0.00E+00	0	0
glutarates	3	1.00E+00	0.00E+00	0	0
glycerides	3	1.00E+00	0.00E+00	0	0
hexoses	3	1.00E+00	0.00E+00	0	0
purine nucleosides	6	1.00E+00	0.00E+00	0	0
purinones	3	3.60E-05	-1.02E+01	3	-1
pyrimidinones	3	1.00E+00	0.00E+00	1	-1
saturated fatty acids	9	1.00E+00	0.00E+00	0	0
sugar acids	3	1.00E+00	0.00E+00	0	0
sugar alcohols	7	1.00E+00	0.00E+00	0	0

unsaturated fatty acids	4	6.40E-04	-7.35E+00	4	-1

Supplemental Table X: ChemRICH cluster information for 2.0MOI_24HPI.

Cluster name	Cluster size	P-value	Transformed P-value	Altered metabolites	Up/down ratio
amino acids	8	7.70E-02	2.85E+00	2	-1
amino acids, acidic	3	1.00E+00	3.02E+00	2	-1
amino acids, aromatic	3	1.00E+00	0.00E+00	0	0
amino acids, basic	3	1.00E+00	0.00E+00	0	0
amino acids, branched-chain	3	1.00E+00	0.00E+00	0	0
amino acids, cyclic	3	1.00E+00	0.00E+00	1	-1
amino acids, sulfur	5	5.20E-03	0.00E+00	1	-1
dicarboxylic acids	7	1.90E-01	0.00E+00	0	0
dipeptides	3	1.00E+00	0.00E+00	0	0
disaccharides	5	1.00E+00	0.00E+00	1	-1
glutarates	3	1.00E+00	0.00E+00	0	0
glycerides	3	1.00E+00	0.00E+00	0	0
hexoses	3	1.00E+00	0.00E+00	1	-1
purine nucleosides	6	1.00E+00	0.00E+00	0	0
purinones	3	3.60E-05	0.00E+00	0	0
pyrimidinones	3	1.00E+00	0.00E+00	0	0
saturated fatty acids	9	1.00E+00	0.00E+00	1	1
sugar acids	3	1.00E+00	0.00E+00	0	0
sugar alcohols	7	1.00E+00	0.00E+00	1	-1

unsaturated fatty acids	4	6.40E-04	5.08E+00	3	-1

Supplemental Table XI: ChemRICH cluster information for 0.5MOI_36HPI.

Cluster name	Cluster size	P-value	Transformed P-value	Altered metabolites	Up/down ratio
adipates	3	1.00E+00	0.00E+00	1	-1
amino acids	9	4.10E-05	1.01E+01	6	-0.4
amino acids, acidic	3	1.00E+00	0.00E+00	1	-1
amino acids, aromatic	3	1.00E+00	0.00E+00	1	-1
amino acids, basic	3	1.00E+00	0.00E+00	0	0
amino acids, branched-chain	3	1.00E+00	0.00E+00	0	0
amino acids, cyclic	4	1.00E+00	0.00E+00	1	-1
amino acids, sulfur	5	1.00E+00	0.00E+00	2	-1
benzene derivatives	3	1.00E+00	0.00E+00	0	0
biogenic polyamines	4	1.00E+00	0.00E+00	1	-1
cholestenes	4	1.00E+00	0.00E+00	1	1
dicarboxylic acids	5	7.30E-03	4.92E+00	3	-1
dipeptides	5	1.00E+00	0.00E+00	0	0
disaccharides	6	6.00E-02	2.81E+00	3	-0.4
ethanolamines	3	1.00E+00	0.00E+00	0	0
glutarates	3	1.00E+00	0.00E+00	1	-1
glyceric acids	3	1.00E+00	0.00E+00	0	0
glycerides	3	1.00E+00	0.00E+00	0	0
hexosephosphates	6	1.00E+00	0.00E+00	1	1
hexoses	3	1.00E+00	0.00E+00	1	-1
pentoses	3	1.00E+00	0.00E+00	0	0

purine nucleosides	7	1.00E+00	0.00E+00	1	1
purinones	3	1.00E+00	0.00E+00	1	-1
pyrimidine nucleosides	3	1.00E+00	0.00E+00	0	0
pyrimidinones	3	1.00E+00	0.00E+00	0	0
saturated fatty acids	14	3.10E-02	3.47E+00	4	1
sugar alcohols	9	4.50E-02	3.10E+00	3	0.4
unsaturated fatty acids	6	1.00E+00	0.00E+00	0	0

Supplemental Table XII: ChemRICH cluster information for 1.0MOI_36HPI.

Cluster name	Cluster size	P-value	Transformed P-value	Altered metabolites	Up/down ratio
adipates	3	1.00E+00	0.00E+00	4	-1
amino acids	9	1.20E-02	4.42E+00	0	-0.6
amino acids, acidic	3	1.00E+00	0.00E+00	0	0
amino acids, aromatic	3	1.00E+00	0.00E+00	0	0
amino acids, basic	3	1.00E+00	0.00E+00	1	0
amino acids, branched-chain	3	1.00E+00	0.00E+00	1	1
amino acids, cyclic	4	1.00E+00	0.00E+00	1	-1
amino acids, sulfur	5	1.00E+00	0.00E+00	0	-1
benzene derivatives	3	1.00E+00	0.00E+00	1	0
biogenic polyamines	4	1.00E+00	0.00E+00	1	-1
cholestenes	4	1.00E+00	0.00E+00	2	1
dicarboxylic acids	5	7.30E-03	4.92E+00	0	-1
dipeptides	5	1.00E+00	0.00E+00	2	0
disaccharides	6	2.20E-01	1.51E+00	0	-1
ethanolamines	3	1.00E+00	0.00E+00	0	0
glutarates	3	1.00E+00	0.00E+00	0	0
glyceric acids	3	1.00E+00	0.00E+00	0	0
glycerides	3	1.00E+00	0.00E+00	0	0
hexosephosphates	6	1.00E+00	0.00E+00	1	0
hexoses	3	1.00E+00	0.00E+00	0	-1
pentoses	3	1.00E+00	0.00E+00	2	0

purine nucleosides	7	4.20E-02	3.17E+00	1	1
purinones	3	1.00E+00	0.00E+00	0	-1
pyrimidine nucleosides	3	1.00E+00	0.00E+00	0	0
pyrimidinones	3	1.00E+00	0.00E+00	4	0
saturated fatty acids	14	1.80E-01	1.71E+00	4	0.1
sugar alcohols	9	2.70E-02	3.61E+00	1	0.1
unsaturated fatty acids	6	1.00E+00	0.00E+00	4	-1

Supplemental Table XIII: ChemRICH cluster information for 2.0MOI_36HPI.

Cluster name	Cluster size	P-value	Transformed P-value	Altered metabolites	Up/down ratio
adipates	3	1.00E+00	0.00E+00	1	-1.0
amino acids	9	3.90E-03	5.55E+00	5	-0.8
amino acids, acidic	3	1.00E+00	0.00E+00	1	-1.0
amino acids, aromatic	3	1.00E+00	0.00E+00	0	0
amino acids, basic	3	1.00E+00	0.00E+00	0	0
amino acids, branched-chain	3	1.00E+00	0.00E+00	0	0
amino acids, cyclic	4	1.00E+00	0.00E+00	1	-1.0
amino acids, sulfur	5	1.00E+00	0.00E+00	2	-1.0
benzene derivatives	3	1.00E+00	0.00E+00	0	0
biogenic polyamines	4	1.10E-01	2.21E+00	2	-1.0
cholestenes	4	1.00E+00	0.00E+00	0	0
dicarboxylic acids	5	7.30E-03	4.92E+00	2	-1.0
dipeptides	5	1.00E+00	0.00E+00	0	0
disaccharides	6	7.80E-02	2.55E+00	2	-1.0
ethanolamines	3	1.00E+00	0.00E+00	0	0
glutarates	3	1.00E+00	0.00E+00	0	0
glyceric acids	3	1.00E+00	0.00E+00	0	0
glycerides	3	1.00E+00	0.00E+00	1	-1.0
hexosephosphates	6	1.00E+00	0.00E+00	0	0
hexoses	3	1.00E+00	0.00E+00	1	-1.0
pentoses	3	1.00E+00	0.00E+00	0	0

purine nucleosides	7	1.00E+00	0.00E+00	1	1
purinones	3	1.00E+00	0.00E+00	1	-1.0
pyrimidine nucleosides	3	1.00E+00	0.00E+00	0	0
pyrimidinones	3	1.00E+00	0.00E+00	0	0
saturated fatty acids	14	7.00E-02	2.66E+00	2	0.1
sugar alcohols	9	4.50E-02	3.10E+00	2	0.1
unsaturated fatty acids	6	5.30E-03	5.24E+00	3	-1.0
